# Chondrocytes-Specific Expression of Osteoprotegerin Modulates Osteoclast Formation in Metaphyseal Bone

**DOI:** 10.1038/srep13667

**Published:** 2015-09-02

**Authors:** Baoli Wang, Hongting Jin, Bing Shu, Ranim R. Mira, Di Chen

**Affiliations:** 1 Key Lab of Hormone and Development (Ministry of Health), Metabolic Diseases Hospital and Tianjin Institute of Endocrinology, Tianjin Medical University, Tianjin 300070, China; 2 Department of Orthopaedics, Center for Musculoskeletal Research, University of Rochester School of Medicine, Rochester, NY 14642, USA; 3 Department of Biochemistry, Rush University Medical Center, Chicago, IL 60612, USA

## Abstract

Bone marrow stromal cells/osteoblasts were originally thought to be the major player in regulating osteoclast differentiation through expressing RANKL/OPG cytokines. Recent studies have established that chondrocytes also express RANKL/OPG and support osteoclast formation. Till now, the *in vivo* function of chondrocyte-produced OPG in osteoclast formation and postnatal bone growth has not been directly investigated. In this study, chondrocyte-specific *Opg* transgenic mice were generated by using type II collagen promoter. The *Col2-Opg* transgenic mice showed delayed formation of secondary ossification center and localized increase of bone mass in proximal metaphysis of tibiae. TRAP staining showed that osteoclast numbers were reduced in both secondary ossification center and proximal metaphysis. This finding was further confirmed by *in vitro* chondrocyte/spleen cell co-culture assay. In contrast, the mineral apposition rates were not changed in *Col2-Opg* transgenic mice. TUNEL staining revealed more apoptotic hypertrophic chondrocytes in the growth plate of *Col2-Opg* mice. Flow cytometry analysis showed fewer RANK-expressing cells in the marrow of *Col2a1-Opg* mice, suggesting the role of OPG in blocking the differentiation of early mesenchymal progenitors into RANK-expressing pre-osteoclasts. Our results demonstrated that OPG expression in chondrocyte increases bone mass in the proximal metaphysis of tibiae through negative regulation of osteoclast formation.

The growth plate chondrocytes are a major player in the process of endochondral ossification that contributes to the longitudinal growth of the skeleton. This process is initiated when progenitor cells in the resting zone are stimulated to proliferate and then proceed through stages of maturation and hypertrophy^1^. Hypertrophic chondrocytes produce a matrix that undergoes calcification, forming a calcified cartilaginous template for new bone formation. Hypertrophic chondrocytes also undergo apoptosis at the lower region of the hypertrophic zone as new blood vessels begin to invade the calcified cartilage and bring in osteoblast precursors, forming trabecular bone^2^,^3^. Although the principles and overall mechanisms of endochondral bone formation are well established, many details remain to be defined. For example, the mechanisms how chondrocytes signal to adjacent osteoclasts (or chondroclasts) to trigger bone resorption remains poorly understood.

It has been established that osteoclast formation and activation require two molecules: macrophage colony-stimulating factor (M-CSF) and RANKL. Both of them are necessary to activate gene transcription to allow osteoclast differentiation^4^. As a member of TNF super family, RANKL is originally believed to be produced by osteoblast lineage cells and activated T-cells and functions differently from M-CSF. While M-CSF increases the pool of osteoclast precursors, RANKL binds to its receptor RANK expressed on osteoclast precursors and mature osteoclasts, signaling to downstream molecules leading to enhanced osteoclast differentiation and activation and reduced apoptosis^4^,^5^. RANK, the receptor of RANKL, is a member of the TNF receptor superfamily^6^. *In vivo* data showed that deletion of either *Rank* or *Rankl* in mice resulted in the same phenotypes, including the profound defects in osteoclast formation, lymph-node formation, and B-cell development^4^,^7^,^8^,^9^,^10^. Osteoprotegerin serves as the decoy receptor of RANKL. It is an atypical member of the TNF receptor superfamily as it is a secreted protein without transmembrane domain. It contains four homologous domains and competes with RANK for binding the same target RANKL, but does not transmit the signal to the osteoclast lineage cells, thus exerting an inhibitory effect on osteoclast differentiation and activation^11^,^12^,^13^,^14^. In support of this, *Opg* knockout mice displayed severe osteoporosis, whereas *Opg* overexpressing mice showed osteopetrosis^11^,^12^,^13^,^14^. The originally identified cells that express OPG include osteoblasts, endothelial cells, vascular smooth muscle, and lymphoid cells^11^,^12^,^13^,^14^. Recently published immunohistochemistry (IHC) and *in situ* hybridization data have shown that RANKL and OPG are also expressed by hypertrophic chondrocytes in murine and rat growth plates^15^,^16^. Further studies demonstrate a novel role for chondrocytes in supporting osteoclast formation by expressing RANKL, an effect regulated by 1, 25-(OH)_2_ vitamin D_3_ and BMP-2^15^,^17^. However, the role of chondrocyte-produced OPG in regulation of osteoclast formation was not implicated in the previous studies.

We have recently reported that inactivation of canonical Wnt/β-catenin signaling in chondrocytes decreases proximal metaphyseal bone mass through enhancing osteoclast formation, which is mediated by the down-regulation of OPG and up-regulation of RANKL^18^. To investigate the role of chondrocyte-produced OPG in osteoclast formation and postnatal bone growth, we generated chondrocyte-specific *Opg* transgenic mice using a 1.0-kb type II collagen promoter (*Col2a1*). Our results demonstrated that chondrocyte-produced OPG plays a significant role in regulation of osteoclast formation during bone growth and bone remodeling.

## Results

### *Opg* transgene was expressed in chondrocytes in *Col2-Opg* transgenic mice

**Figure 1:**
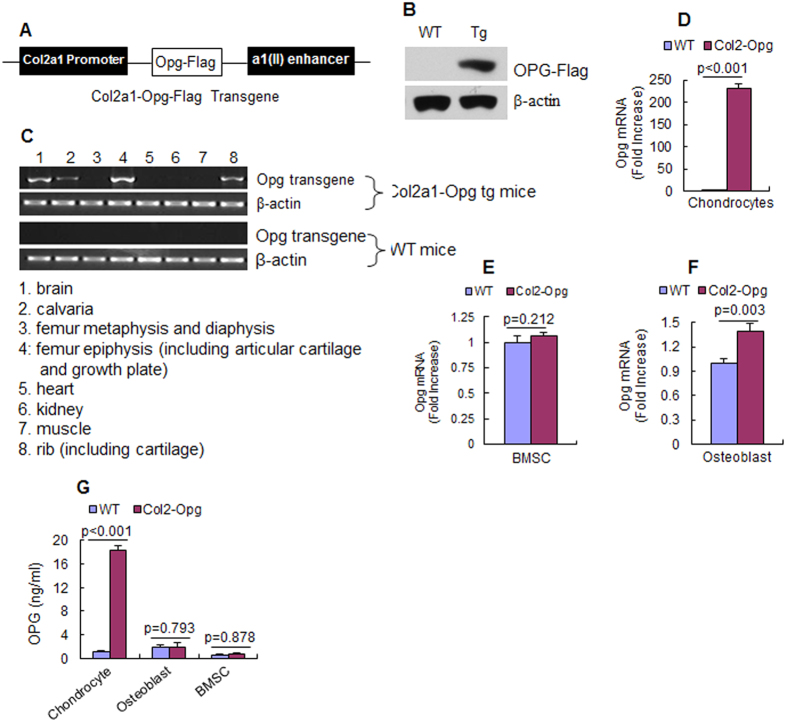
*Opg-Flag* transgene was expressed in chondrocytes in *Col2-Opg* mice. (**A**) The diagram shows the *Col2-Opg* transgene construct. (**B**) Expression of OPG-Flag protein was detected in chondrocytes derived from *Col2-Opg* transgenic mice by Western blot analysis using the anti-Flag antibody. (**C**) Expression of *Opg-Flag* mRNA was examined in multiple tissues by RT-PCR using the transgene specific primers. (**D**–**G**) Expression levels of *Opg* mRNA and protein in primary sternal chondrocytes, calvarial pre-osteoblasts and bone marrow stromal cells were examined. Data are presented as means ± SD (n = 3–4 per group).

In this study, we generated *Col2-Opg* transgenic mice in which expression of the *Opg-Flag* transgene was targeted to chondrocytes using the 1.0 kb type II collagen promoter (*Col2a1*) (Fig. 1A)^19^,^20^,^21^. Two independent lines of *Col2-Opg* transgenic mice were established and both of them displayed similar phenotypes. *Col2-Opg* transgenic mice are viable, fertile with normal body size and have no any gross, physical, or behavioral abnormalities.

Expression of the OPG-Flag protein was detected by Western blotting using an anti-Flag antibody in primary sternal chondrocytes isolated from *Col2-Opg* transgenic mice but not in those from WT mice (Fig. 1B). To determine the specificity of the *Opg-Flag* transgene expression, total RNA was extracted from multiple tissues and expression of *Opg-Flag* mRNA was examined by RT-PCR using transgene specific primers (Opg Fw and Flag-Rev). Strong expression of *Opg-Flag* was detected in femur epiphysis that spans from the articular surface to the lower border of the growth plate. Weaker expression of the transgene was detected in other bone tissues involving cartilages like calvaria and rib and in the brain, but was not detected in other tissues, including heart, kidney, and muscle (Fig. 1C). Interestingly, the transgene was not detected in femur metaphysis and diaphysis that were free of cartilages. This is consistent with our previous findings in *Col2CreER*^*T2*^ transgenic mice^18^,^22^. We also cultured the primary sternal chondrocytes, calvarial pre-osteoblasts and bone marrow stromal cells (BMSCs) and compared the mRNA and protein expression of *Opg* in the *Col2-Opg* transgenic mice to that in the WT control littermates (Fig. 1D–G). The results showed that the *Opg* mRNA expression in chondrocytes was increased by 232-fold in the *Col2-Opg* transgenic mice (Fig. 1D). ELISA measurement of chondrocyte conditioned medium also revealed a significant increase in the OPG protein levels in *Col2-Opg* mice (Fig. 1G). In contrast, the mRNA expression of *Opg* did not increase in BMSCs and only increased by 40% in calvarial pre-osteoblasts (Fig. 1E,F) in *Col2-Opg* mice. No significant change of OPG protein level was observed in either primary calvarial pre-osteoblasts or BMSCs (Fig. 1G). Moreover, the serum OPG was increased by 73% in the 5-week old transgenic mice^18^.

### Expression of *Opg* transgene in chondrocytes increased trabecular bone mass

**Figure 2:**
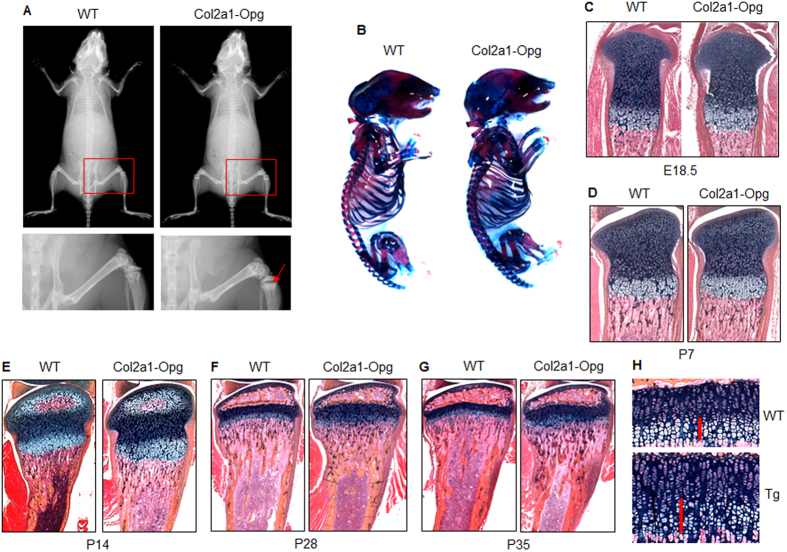
X-ray radiographic and histological analyses of *Col2-Opg* mice. (**A**) The radiodensity of long bones of 5-week-old *Col2-Opg* mice was examined by X-ray (red arrows: primary spongiosa area). (**B**) E18.5 embryos were stained with Alcian blue/Alizarin red. (**C**–**G**) Histological staining of tibial sections was performed in E18.5, P7, P14, P28 and P35 WT and *Col2-Opg* mice. The formation of secondary ossification center was delayed (**E**) and the hypertrophic zone was enlarged (**F** and **G**) in *Col2-Opg* mice. (**H**) The thickness of the growth plate was measured in P35 mice (red bars: length of hypertrophic zone).

We then examined changes in bone mass of the transgenic mice by X-ray radiographic, histological and μCT analyses. X-ray radiographic results showed that the radiodensity in the proximal metaphyseal regions of tibiae was higher in the 5-week-old *Col2-Opg* mice than in their WT littermates. In contrast, the radiodensity in the mid-shaft of the bones was not changed (Fig. 2A). We then examined if there are defects in bone or cartilage development in the embryonic stage. Alcian blue/Alizarin red staining of E18.5 skeleton revealed no significant differences between WT and *Col2a1-Opg* embryos (Fig. 2B).

Histological sections were stained with Alcian blue/Hematoxylin & Orange G. The *Col2-Opg* mice did not show significant changes in bone mass at E18.5 embryos (Fig. 2C) and P7 mice (Fig. 2D). The *Col2-Opg* mice had normal columnar arrangement of the growth plate chondrocytes. However, the *Col2-Opg* mice showed delay in the formation of the secondary ossification center. At P14 in WT mice, mineralized cartilage in secondary ossification centers has been removed and replaced by trabecular bone; in contrast, mineralized regions have only partly been replaced by trabecular bone in *Col2-Opg* mice. The trabecular bone volume in the metaphyseal region was not significantly altered (Fig. 2E). At P28 and P35, the trabecular bone volumes in the proximal metaphyseal region were significantly increased in *Col2-Opg* mice (Fig. 2F,G). The transgenic mice maintained the typical columnar structure of growth plate chondrocytes, but the thickness of the growth plate, especially the hypertrophic zone, was 60% greater in the transgenic mice than in WT controls (Fig. 2H).

**Figure 3:**
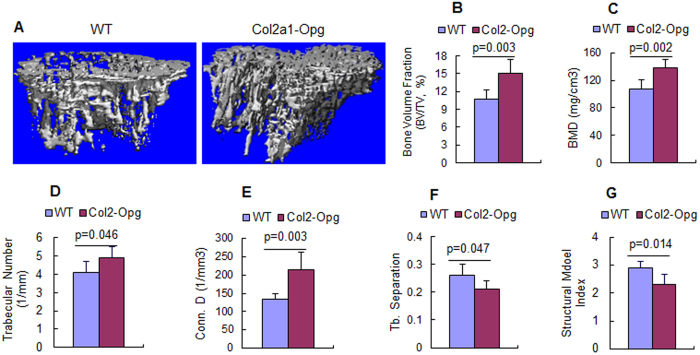
μCT analysis of the bone mass of *Col2-Opg* mice. (**A**) μCT analysis of bone mass of the proximal metaphysis of tibiae was examined in 4-week-old *Col2-Opg* mice and WT littermates. (**B**–**G**) Histomorphometric parameters, including bone volume (BV/TV, %), bone mineral density (BMD), trabecular number (Tb.N.), connectivity density (Conn. **D**.), trabecular separation (Tb.Sp.), and structure model index (SMI), were analyzed in *Col2-Opg* mice and WT littermates. Data are represented as means ± SD (n = 6 per group).

Consistent with this, μCT imaging revealed the bone mass increase in the proximal metaphysis beneath the growth plate in 4-week-old *Col2-Opg* mice (Fig. 3A). The trabecular bone volume (% BV/TV) was 41% greater (Fig. 3B) and the bone mineral density was 29% greater (Fig. 3C) in *Col2-Opg* mice. The trabecular number (Tb. N., 1/mm) was 20% higher (Fig. 3D), and the connectivity density (Conn. D.) was 60% higher (Fig. 3E), while the trabecular separation (Tb.Sp.) was 24% lower (Fig. 3F) in *Col2-Opg* mice. The structural model index (SMI, a measure of the shape of trabeculae; 0 for plates and 3 for cylindrical rods) was significantly decreased by 21% in *Col2-Opg* mice (Fig. 3G).

### Osteoclast formation was altered in *Col2-Opg* mice

**Figure 4:**
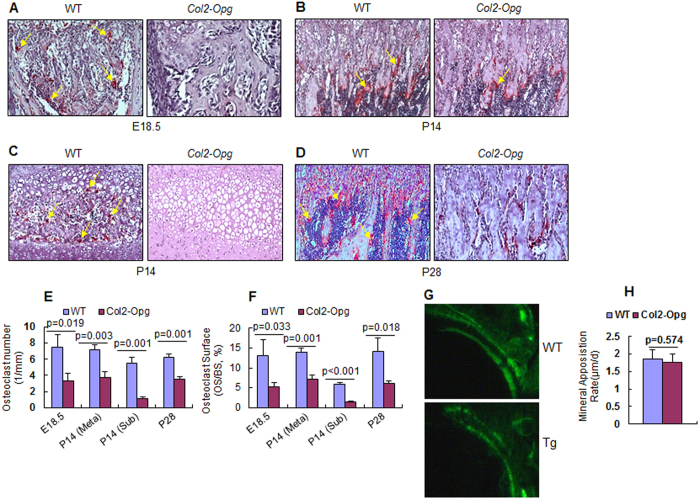
Osteoclast formation was reduced in *Col2-Opg* mice. (**A**–**D**) TRAP staining in the proximal metaphysis of tibiae was performed in E18.5 embryos, P14 and P28 mice (yellow arrows: TRAP-positive multi-nuclear osteoclasts). In P14 mice, TRAP staining was performed in the secondary ossification center area (**C**). (**E**,**F**) Histomorphometric analysis of osteoclast numbers and osteoclast surface was performed. (**G**,**H**) The mineral appositional rates (MAR) were measured in WT and *Col2-Opg* mice. Data are presented as means ± SD (n = 3–5 per group).

We performed the TRAP staining in tibiae from E18.5 embryos and P14 and P28 postnatal mice. The numbers of TRAP-positive osteoclasts and osteoclast surfaces were significantly decreased in *Col2-Opg* mice at all these time points (Fig. 4A–F). Of note, in 14-day-old *Col2-Opg* mice, the osteoclast numbers and osteoclast surfaces were lower not only in the primary metaphyseal region (Fig. 4B,E,F), but also in the secondary ossification center region, where the osteoclast numbers and osteoclast surfaces were reduced by 79% and 75%, respectively, compared to WT controls (Fig. 4C,E,F). This may explain why the formation of the secondary ossification center was delayed in *Col2-Opg* transgenic mice. We also examined changes in mineral apposition rate (MAR), the indicator of osteoblast activity, and found that the MAR was not significantly changed in *Col2-Opg* mice (Fig. 4G,H).

### *In vitro* osteoclast formation was reduced in *Col2-Opg* mice

**Figure 5:**
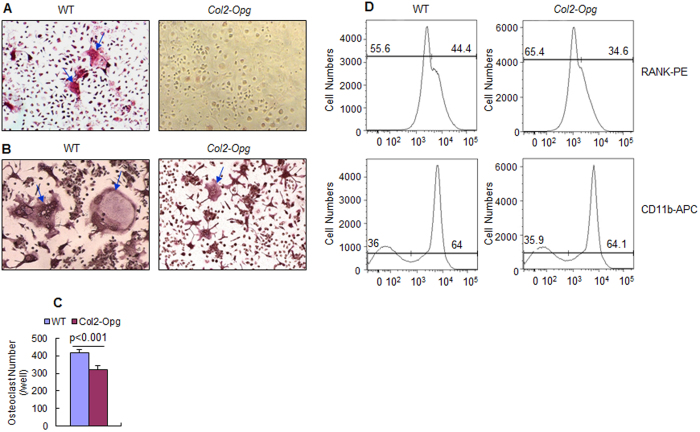
Osteoclast differentiation was reduced in *Col2-Opg* mice. (**A**) Primary chondrocytes derived from WT and *Col2-Opg* mice were co-cultured with WT spleen cells in the presence of 10^−8^ M 1,25-(OH)_2_ D_3_ (blue arrows: multinuclear osteoclasts). (**B**,**C**) Osteoclast formation was also studied in bone marrow cells derived from Col2a1-Opg transgenic mice in the presence of 50 ng/ml sRANKL and 10 ng/ml M-CSF. (**D**) Flow cytometry analysis was performed using monoclonal antibodies against RANK and CD11b (n = 3 per group). Data are presented as means ± SD.

To determine the role of chondrocyte-produced OPG in osteoclast formation, we performed chondrocyte-spleen cell co-culture experiments in the presence of 10^–8^ M 1,25-(OH)_2_ vitamin D_3_, TRAP-positive osteoclast formation was completely abolished when spleen cells were cultured with chondrocytes derived from *Col2-Opg* mice (Fig. 5A). To further identify if the overexpression of the *Opg* transgene alters the osteoclast formation from bone marrow cells, we cultured bone marrow cells in the presence of M-CSF and RANKL. Osteoclast formation was significantly reduced when bone marrow cells derived from *Col2-Opg* transgenic mice were cultured for osteoclast formation assay (Fig. 5B,C), suggesting that bone marrow cells were affected in chondrocyte-specific *Col2-Opg* mice. The *Opg* overexpression might have changed bone marrow cell populations. To examine this, we performed flow cytometric analysis using monoclonal antibodies against RANK and CD11b. The results showed that the RANK positive cells were reduced in *Col2-Opg* mice compared to WT mice; while the CD11b positive cells did not change significantly in *Col2-Opg* mice (Fig. 5D). These results suggest that RANK-expressing pre-osteocalsts may also be involved in OPG-mediated osteoclast inhibition.

### Changes in growth plate chondrocyte function in *Col2-Opg* mice

**Figure 6:**
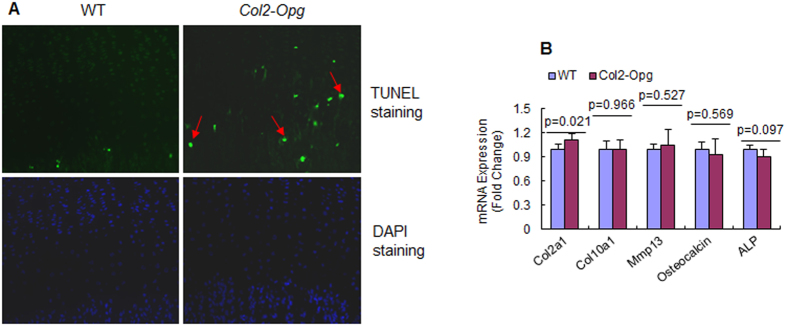
Changes in growth plate chondrocyte function in *Col2-Opg* mice. (**A**) TUNEL staining was performed using 4-week-old WT and *Col2-Opg* mice (red arrows: apoptotic chondrocytes). (**B**) Real-time PCR was performed to determine changes in expression of *Col2a1*, *Col10a1*, *Mmp13*, *ostocalcin*, and *Alp* in sternal chondrocytes derived from WT and *Col2-Opg* mice. Data are presented as means ± SD (n = 3 per group).

To evaluate chondrocyte apoptosis, we performed TUNEL staining in 4-week-old WT and *Col2-Opg* mice and counted percentage of green fluorescent cells by normalizing to DAPI-positive cells. We found that the number of apoptotic hypertrophic chondrocytes was significantly increased in *Col2-Opg* mice (Fig. 6A). This result suggests that the expanded width of the hypertrophic zone could be owing to fewer chondrocytes being resorbed by osteoclasts after entering the apoptosis phase.

To further clarify if OPG affects chondrocyte development, mRNA expression of several chondrocyte marker genes were examined by real-time PCR assay. Expression levels of *collagen type II (Col2a1), collagen type X* (*Col10a1*), *matrix metalloproteinase 13* (*Mmp13*), *osteocalcin (OC)*, and *alkaline phosphatase (Alp)* were not significantly altered in *Col2-Opg* chondrocytes (Fig. 6B). These findings demonstrated that the OPG does not play a significant role in regulation of chondrocyte maturation.

### Slight changes in osteoblast and adipocyte formation in *Col2-Opg* mice

**Figure 7:**
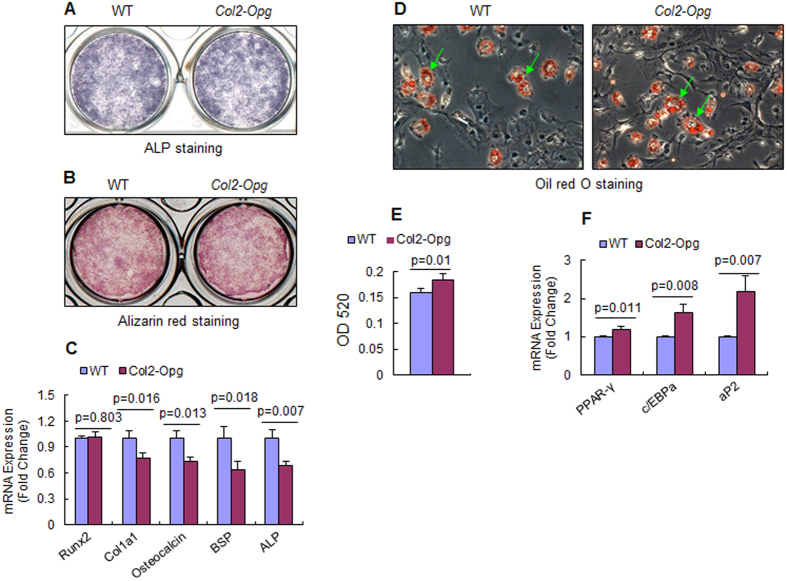
Osteoblast and adipocyte differentiation was slightly altered in *Col2-Opg* mice. (**A**,**B**) ALP and Alizarin red O staining was performed using BMSCs from WT and *Col2-Opg* mice. (**C**) Real-time PCR was performed to examine expression of osteoblast marker genes. (**D**) Oil red O staining was performed to determine adipocyte differentiation from BMSCs derived from WT and *Col2-Opg* mice (green arrows: Oil red O staining-positive cells). (**E**) Real-time PCR was performed to examine expression of adipocyte marker genes. Data are presented as means ± SD (n = 4 per group).

We then examined osteoblast differentiation in BMSCs of *Col2-Opg* mice. ALP and Alizarin red staining showed similar osteoblast differentiation tendency in *Col2-Opg* mice compared to WT controls (Fig. 7A,B). Interestingly, results of real-time PCR assay revealed that the mRNA expression of *Runx2* was not changed, while expression levels of Col1a1, Alp, OC and Bsp were slightly reduced (*p* < 0.05) when BMSCs of *Col2-Opg* mice were cultured with osteoblast differentiation medium (Fig. 7C). These results likely reflect the coupling between osteoclast-mediated resorption and osteoblast-mediated bone formation.

We also examined *in vitro* adipocyte formation and adipocyte marker gene expression. When cells were cultured with adipogenic medium, mild increase in adipocyte numbers was observed from BMSCs of *Col2-Opg* mice, measured by Oil red O staining (Fig. 7D,E). An up-regulation of *C*/*EBPα* and *aP2* mRNA expression in BMSCs from transgenic mice was observed whereas *PPARγ* mRNA was not significantly changed (Fig. 7F).

## Discussion

OPG is known to be produced by osteoblasts and BMSCs in bone. Recent studies reported that RANKL and OPG are also expressed in growth plate chondrocytes^15^,^22^,^23^,^24^,^25^. RANKL/OPG expressed in chondrocytes may modulate osteoclast (or chondroclast) differentiation by a mechanism similar to osteoblast-dependent osteoclastogenesis. So far, several groups have reported the *in vitro* and *in vivo* chondrocyte-dependent osteoclastogenesis^16^,^17^,^18^. This implicates that the growth plate chondrocytes might be the direct regulator of bone mass at the chondro-osseus junction region. Our group further demonstrated that bone morphogenetic protein-2 enhances osteoclast formation by promoting RANKL expression in chondrocytes via Runx2/Smad1 pathway^16^.

The crucial role of OPG in bone modeling and remodeling has been demonstrated in animal models using conventional deletion or non-tissue specific overexpression strategies. Targeted deletion of *Opg* in mice results in severe, early-onset osteoporosis due to excessive osteoclastogenesis^11^,^12^,^13^,^26^. On the contrary, transgenic mice overexpressing *Opg* in the liver have high levels of OPG protein in their circulation and exhibit a marked increase in bone density. Recently, we created chondrocyte specific *β-catenin* conditional KO mice showing severe bone loss due to the enhanced osteoclast formation in proximal metaphysis. Detail *in vitro* investigations demonstrated that the β-catenin signaling in chondrocytes inhibits osteoclast formation by up-regulation of OPG and down-regulation of RANKL expression in chondrocytes^18^.

Several recent reports suggest that *Col2a1, Col10a1* and *Aggrecan* promoters could drive Cre recombinase expression in hypertrophic chondrocytes during embryonic and postnatal skeletal development. A subset of the Cre targeting chondrocyte population could transform into osteoblast precursors or osteoblast-like cells^27^,^28^,^29^,^30^,^31^. To determine the targeting efficiency and specificity of *Col2-CreER* mice, we have bred these mice with *Rosa*^*mT/mG*^ reporter mice and found that some of *Col2*-positive cells could be detected at primary spongiosa area underneath the growth plate, suggesting that these cells may be derived from hypertrophic chondrocytes^18^,^28^. These cells could directly contact osteoclast precursors and promote osteoclast formation and differentiation. Although a subset of chondrocyte-derived cells in primary spongiosa area could contribute to osteoclast formation, our ELISA assay revealed over 10-fold increase in OPG protein by *in vitro* cultured chondrocytes isolated from the *Col2-Opg* transgenic mice, suggesting that chondrocytes are the major source of OPG production in *Col2-Opg* transgenic mice and may play a major role in regulation of osteoclast formation.

Comparing to the generalized osteopetrosis phenotype observed in the liver-specific *Opg* transgenic mice, our *Col2-Opg* mice showed that bone mass increase is only localized to the proximal metaphysis in *Col2-Opg* mice. This may represent local OPG production by chondrocytes without systemic effect of OPG in *Col2-Opg* mice. At P28 and P35, the trabecular bone volume in the proximal metaphyseal region was greater in the *Col2-Opg* transgenic mice than in the WT littermates. This is due to the reduction of osteoclast formation in transgenic mice. The thickness of the growth plate, especially the hypertrophic zone, was increased in transgenic mice as a result of the reduced osteoclast formation. TUNEL staining results showed that numbers of apoptotic hypertrophic chondrocytes were significantly increased in *Col2-Opg* transgenic mice, suggesting that the expanded width of hypertrophic zone could be owing to fewer chondrocytes being resorbed by osteoclasts after entering the apoptosis phase. However, transgenic mice maintained the typical columnar arrangement of growth plate chondrocytes, suggesting that OPG protein does not alter the biological phenotype and behavior of growth plate chondrocytes. We did not observe significant changes in bone mass in older mice and this is probably due to reduced *Col2a1* promoter activity in older mice.

*In vitro* chondrocyte-spleen cell co-culture experiment demonstrated that the *Opg-*overexpressing chondrocytes inhibited WT spleen cells to form TRAP-positive osteoclast. However, by then it still remained unknown if *in vivo* the overexpression of the *Opg* transgene in chondrocytes changed the percentage of osteoclast lineage in bone marrow. Thus, we cultured bone marrow cells and found that osteoclast formation was significantly reduced with bone marrow cells derived from *Col2a1-Opg* transgenic mice, suggesting that bone marrow cells were affected in *Col2a1-Opg* mice. The *Opg* transgene might have changed cell populations of bone marrow. To further confirm this, we performed flow cytometric analysis and revealed that the *Col2a1-Opg* mice have fewer RANK positive cells but similar percentage of the CD11b positive cells compared to the WT littermates. This suggests that OPG is able to down-regulate the RANK expression in mononucleated cells of bone marrow or decrease the number of RANK-expressing cells. This is interesting because it is previously not known if RANKL/OPG has direct regulatory effect on RANK expression until recent report showing that RANKL induces RANK expression in bone marrow cells and RAW264.7 cells before the formation of osteoclasts^32^. Our present study provides the *in vivo* data that support this finding. Thus it is clear that RANKL/OPG acts on not only RANK-expressing preosteoclasts, but early mesenchymal progenitor cells as well to trigger/block the differentiation of osteoclasts.

In our study, decreased osteoclast formation were not accompanied by dramatic changes in osteoblastic activities in the *Opg-*overexpressing tibial epiphyses, as indicated by the results of calcein double labeling, which did not reveal any change in MAR in *Col2a1-Opg* mice. This disagrees with Bucay’s study in which they reported the presence of abundant calcein deposition in the *Opg*^*−/−*^ mice^11^,^12^,^13^,^14^, indicating that bone mineralization was enhanced and osteoblast activity was increased in their mutant mice. Our findings suggest that the locally produced OPG protein by the growth plate chondrocytes mainly affects adjacent osteoclasts (or chondroclasts) and does not contribute much to bone remodeling. To further identify the events at the cellular and molecular level, we examined osteoblast differentiation from bone marrow cells. Although ALP and Alizarin red staining showed similar osteoblast differentiation tendency in bone marrow cells from the transgenic mice, real-time PCR analysis revealed that the mRNA expression of *Col1a1, osteocalcin*, and *bone sialoprotein* (*Bsp*) was mildly reduced in bone marrow cells from *Col2a1-Opg* mice, likely reflecting the coupling between osteoclast-mediated resorption and osteoblast-mediated bone formation. Consistent with this, when bone marrow cells were cultured under adipogenic conditions, adipocyte formation and the mRNA expression of adipogenic genes, including C/EBPα and aP2, were slightly increased in the *Col2-Opg* transgenic mice. The significance of regulation of osteoblast and adipocyte formation by chondrocyte produced OPG needs to be further investigated.

In this study we overexpressed *Opg* transgene in *Col2*-expressing chondrocytes. Although this is an overexpression study, it clearly demonstrates that chondrocyte produced OPG could regulate osteoclast formation *in vivo*. This study also provides evidence that pathological upregulation of OPG expression in chondrocytes could lead to osteopetrosis phenotype in metaphyseal bone.

## Methods

### Generation of *Col2-Opg* transgenic mice

To generate *Col2a1-Opg* transgenic mice, a DNA fragment encoding murine osteoprotegerin fused with a 3′ Flag tag was cloned into the *Not*I site of an expression vector PKN185 in which the transgene expression is under the control of *Col2a1* promoter and enhancer^33^,^34^. The expression cassette of *Opg-Flag* was excised by *Nde*I and *Hin*dIII digestion. The *Opg-Flag* transgene was then purified and injected into pronuclei of fertilized eggs. Positive transgenic founder mice were identified by PCR and confirmed by Southern blot analysis. The sequences of PCR primers for genotyping *Col2-Opg* mice are as follows: Fw, 5′- GAGCATATAACTGGAGCCTC -3′ and Rev, 5′- CACACAGGGTGACATCTATTC -3′. The 1120-bp PCR product was detected in the transgenic mice. The animal protocol and experimental methods were approved by the Institutional Animal Care and Use Committee (IACUC) of University of Rochester (New York) and all animal experiments were carried out in accordance with the approved protocol and guidelines.

### Micro-CT analysis

Quantitative analysis of bone samples was performed in tibiae of 5-week-old *Col2-Opg* transgenic mice and their WT littermates at 10 μM resolution on a micro-CT Scanner (VivaCT 40; Scanco Medical AG, Bassersdorf, Switzerland) as previously described^35^.

### Histology

Histological analyses were performed in tibiae from *Col2a1-Opg* transgenic mice and their WT littermates. To reveal patterning changes in cartilage and bone of the entire skeleton, whole embryos (E18.5) were sequentially stained with Alcian blue (for cartilage) and Alizarin red (for bone) as described in previous studies^36^. In short, after fixation with 95% ethanol for 48–72 hours, embryos were stained with 0.03% (w/v) Alcian blue in 80% ethanol and 20% acetic acid solution for 1–3 days, followed by 0.03% (w/v) Alizarin red in 1% KOH solution for another 12–24 hours, and then maintained in a solution of 2% KOH:glycerol (20:80) until analysis.

To reveal bone mass changes prenatally and postnatally, bone samples were dissected, fixed in 10% formalin, decalcified in 5% formic acid or 14% EDTA, and embedded in paraffin. Serial sections of tibiae were cut every 3 μm from both the medial and lateral compartments. The formic acid-treated sections were stained with Alcian blue/Hematoxylin and counterstained with Orange G to evaluate the structures and volumes of cartilage and bone. The EDTA-treated sections were stained for tartrate acid resistant phosphatase (TRAP) and counterstained with Hemotoxylin. Histomorphometric analyses were performed on proximal tibial metaphyses to quantify the osteoclast numbers and osteoclast surfaces using the OsteoMeasure system (OsteoMetrics, Atlanta, GA). Osteoclast numbers and osteoclast surfaces were expressed per mm in longitudinal sections.

The mineral apposition rate (MAR) was determined by calcein double labeling. *Col2a1-Opg* transgenic mice and their WT littermates were injected with calcein (10 mg/kg) 7 days and 2 days prior to sacrifice. Following dissection, calvariae and tibiae were fixed in 70% ethanol and embedded in polymethyl methacrylate resin for sectioning. Coronal sections of comparable anatomic position were examined by fluorescence microscopy. The mean distance between the calcein double labels was measured using AxioVision 3.0 image software.

### Enzyme-linked immunosorbent assay (ELISA)

Blood was collected right before sacrifice and the serum was prepared. The levels of mouse OPG were measured using an ELISA kit (RND systems, Minneapolis, MN). The conditioned medium was collected from cultures of primary sternal chondrocytes, calvarial osteoblasts and bone marrow stromal cells and subjected to ELISA assays, following the manufacturer’s recommendation.

### Chondrocyte/spleen cell co-culture assay

3-day-old neonatal mice were genotyped and sacrificed. Primary mouse sternal chondrocytes were isolated from the anterior rib cage of 3-day-old neonatal mice as described previously^36^,^37^. Spleen cells were isolated from 2-month-old WT mice by disaggregation through a wire sieve and seeded in a 48-well culture plate at the density of 4 × 10^5^ cells/well. Primary sternal chondrocytes were seeded into the culture at the density of 2,000 cells/well and co-cultured with the spleen cells for 2 days in minimum essential medium-α (α-MEM) supplemented with 10% fetal bovine serum (FBS) in the presence of 20 ng/ml M-CSF (R&D Systems, Minneapolis, MN, USA). Cells were then treated with 20 ng/ml M-CSF and 10^−8^ M 1,25-(OH)_2_ vitamin D_3_ (Sigma, St. Louis, CA, USA) for additional 5 days. The adherent cells were fixed and stained for TRAP, a marker of mature osteoclasts. The numbers of TRAP-positive cells with 3 or more nuclei were counted.

### Osteoclast formation from bone marrow

Bone marrow cells were isolated from 4-week-old mice according to the previously published protocol^35^. The cells were seeded in a 48-well plate at the density of 5 × 10^5^/well in minimum essential medium-α (α-MEM) supplemented with 10% fetal bovine serum (FBS). The cells were treated with M-CSF (20 ng/ml) for 2 days and then cultured in the presence of 20 ng/ml M-CSF and 50 ng/ml recombinant human sRANKL (R&D Systems, Minneapolis, MN, USA) for additional 5 days. TRAP staining was performed and the TRAP-positive multi-nucleated osteoclasts were counted.

### *In vitro* osteoblast formation assay

Bone marrow cells were isolated from femurs and tibiae of 4-week-old mice and cultured with α-MEM supplemented with 10% FBS in a 10-cm culture dish. When cells reached 80% confluence, they were digested with 0.25% trypsin and 0.02% EDTA. Cells were plated into 6 well culture plates at the density of 4 × 10^5^ cells/well in α-MEM containing 10% FBS, 50 μg/mL ascorbic acid and 5 mmol/L β-glycerophosphate. Cells were cultured for 7 days followed by alkaline phosphatase (ALP) staining, or 21 days followed by Alizarin red staining (nodule formation assay), or 3 days followed by RNA extraction and real-time RT-PCR assay^35^,^38^,^39^.

### Oil red O staining

Fully differentiated adipocytes were gently washed twice with phosphate-buffered saline (PBS), and then fixed in 4% paraformaldehyde for 10 min. The samples were then washed twice with deionized water, and 60% saturated oil-red O staining was carried out for 5 min. For oil-red O quantification, 4% IGEPAL CA 630 (Sigma) in isopropanol was added to each well. Light absorbance was measured at 520 nm.

### RNA extraction and real-time PCR assay

Total cellular and tissue RNA was prepared by PureLink™ RNA Mini Kit (Invitrogen, Carlsbad, CA) according to the manufacturer’s protocol. 1 μg of total RNA was used to synthesize cDNA using iScripts cDNA Synthesis Kit (Bio-Rad, Hercules, CA). Real-time PCR amplifications were performed using specific primers and SYBR green real-time PCR kit. The primers used for the PCR are listed in Table 1. The thermal cycling conditions were as follows: an initial denature at 95 °C for 3 minutes, followed by 40 cycles of 20 seconds of denature at 95 °C, 20 seconds of annealing at 58 °C, and 20 seconds of extension at 72 °C. The levels of the target gene expression were normalized to that of *β-actin* in the same cDNA sample.

### Transferase-mediated digoxigenin-dUTP nick-end labeling (TUNEL)

A TUNEL staining kit (DeadEnd Fluorometric TUNEL System, Promega, Madison, WI) was used to assess cell apoptosis by catalytically incorporating fluorescein-12-dUTP at 3′-OH DNA ends using the terminal deoxynucleotidyl transferase and recombinant enzyme (rTDT).

### Flow cytometry analysis

BMSCs were isolated from 4-week-old mice. 1 × 10^6^ cells per sample were collected and fixed with 1% paraformaldehyde (15 min at 4 °C), permeabilized with 0.05% Tween 20 (15 min at 37 °C). Cells were first blocked with the Fc blocker in FACS buffer for 15 min, followed by incubation for 20 min on ice with mouse RANK monoclonal antibody (clone 9A725, Thermo Scientific, Rockford, IL) conjugated with PE and rat anti-mouse CD11b antibody conjugated with APC (BD Biosciences, San Jose, CA), and washed by FACS buffer. Data were acquired with a FACSCalibur flow cytometer (BD Biosciences), and analyzed with CellQuest software version 3.5.1 (BD Biosciences).

### Statistical analysis

Data were presented as mean ± standard deviation (SD). Independent samples *t*-test was used to determine statistical difference between two groups using the SPSS program. P values less than 0.05 were considered significant.

## Additional Information

**How to cite this article**: Wang, B. *et al.* Chondrocytes-Specific Expression of Osteoprotegerin Modulates Osteoclast Formation in Metaphyseal Bone. *Sci. Rep.* **5**, 13667; doi: 10.1038/srep13667 (2015).

## Figures and Tables

**Table 1:** Names and sequences of PCR primers.

**Primer name**	**Sequence**
*Opg*, forward	5′- CAGAGCGAAACAC AGTTTG -3′
*Opg*, reverse	5′- CACACAGGGTGACATCTATTC -3′
*Runx2*, forward	5′- CCTGAACTCTGCACCAAGTCCT-3′
*Runx2*, reverse	5′- TCATCTGGCTCAGATAGGAGGG-3′
*Alp*, forward	5′- CCAGAAAGACACCTTGACTGTGG-3′
*Alp*, reverse	5′- TCTTGTCCGTGTCGCTCACCAT-3′
*Col1a1*, forward	5′- CCTCAGGGTATTGCTGGACAAC-3′
*Col1a1*, reverse	5′- CAGAAGGACCTTGTTTGCCAGG-3′;
*OC*, forward	5′- GCAATAAGGTAGTGAACAGACTCC-3′
*OC*, reverse	5′- CCATAGATGCGTTTGTAGGCGG -3′
*BSP*, forward	5′-GAGCCAGGACTGCCGAAAGGAA-3′
*BSP*, reverse	5′-CCGTTGTCTCCTCCG CTGCTGC-3′
*Col10a1*, forward	5′-ACCCCAAGGACCTAAAGGAA-3′
*Col10a1*, reverse	5′-CCCCAGGATACCC TGTTTTT-3′
*Mmp13*, forward	5′-TTTGAGAACACGGGGAAGA-3′
*Mmp13*, reverse	5′-ACTTTGTTGCCAATTCCA GG-3′
*β-actin*, forward	5′- GAACCCTAAGGCCAACCGTG-3′
*β-actin*, reverse	5′-ACCAGAGGCATACAGGGACAG-3′
